# Serum Immunometabolic Biomarkers Reveal Distinct Phenotypes in Chronic Urticaria

**DOI:** 10.3390/diagnostics16081148

**Published:** 2026-04-13

**Authors:** Nilay Duman, Can Muftuoglu, Begüm Tahhan, Tolga Coşkun, Deniz Ece, Ufuk Mert, Sıla Özkal, Ayse Caner

**Affiliations:** 1 Department of Dermatology, Faculty of Medicine, Ege University, Bornova, 35100 İzmir, Türkiye; nilay.duman@ege.edu.tr; 2 Department of Basic Oncology, Institute of Health Sciences, Ege University, Bornova, 35100 İzmir, Türkiye; canmuftuoglu1990@gmail.com (C.M.); tolgacoskun31@gmail.com (T.C.); denizece6716@gmail.com (D.E.); ufuk.mert@ege.edu.tr (U.M.); 3 Faculty of Medicine, Ege University, 35100 İzmir, Türkiye; begumtahhan@gmail.com (B.T.); silaozkall@gmail.com (S.Ö.); 4 Ataturk Vocational School of Health Services, Ege University, 35100 İzmir, Türkiye; 5 Department of Parasitology, Faculty of Medicine, Ege University, Bornova, 35100 İzmir, Türkiye

**Keywords:** chronic urticaria, mast cells, DAO, histamine, lactic acid, sCD14, anti-TPO, metabolic reprogramming, autoimmune phenotype

## Abstract

**Background/Objectives:** Chronic urticaria (CU) is a heterogeneous inflammatory disorder generally attributed to mast cell activation. However, emerging evidence suggests that metabolic reprogramming and systemic immune dysregulation also contribute to the disease pathophysiology. This study aimed to investigate the interplay between epithelial barrier integrity, innate immune regulation, metabolic activity, and mast cell effector mechanisms in CU. **Methods:** Forty CU patients and 40 healthy controls were evaluated. Clinical parameters included disease severity, disease subtype, antihistamine response, IgE levels, anti-TPO status, gastrointestinal symptoms, and angioedema. Serum levels of histamine, intestinal fatty acid-binding protein (IFABP), soluble CD14 (sCD14), diamine oxidase (DAO), D-lactic acid, endotoxin, zonulin, calprotectin, and related ratios were measured. Disease activity and control were assessed using the UAS7 and UCT scores. **Results:** CU patients exhibited significantly higher DAO (*p* = 0.003) and lactic acid (*p* = 0.004) levels compared to controls, whereas other markers showed no significant differences. In anti-TPO-positive patients, sCD14 levels were reduced (*p* = 0.024), while histamine/sCD14 (*p* = 0.005), lactic acid/sCD14 (*p* = 0.014), IFABP/sCD14 (*p* = 0.008), and zonulin/sCD14 (*p* = 0.027) were significantly elevated, suggesting relative amplification of metabolic and barrier-related signals under impaired innate immune regulation. Severe anti-TPO-positive patients exhibited lower sCD14 (*p* = 0.022) and NLR (*p* = 0.013) but higher UAS7 (*p* = 0.032), histamine (*p* = 0.011), calprotectin (*p* = 0.041), and CD14-normalized ratios, including histamine (*p* = 0.003), IFABP (*p* = 0.028), lactic acid (*p* = 0.019), zonulin (*p* = 0.029), and calprotectin (*p* = 0.011) compared with severe anti-TPO-negative patients, indicating a mast cell-dominant and metabolically active inflammatory phenotype. The lactic acid/DAO ratio was significantly lower in controlled versus uncontrolled CU (*p* = 0.013) and showed discriminatory potential for disease control. Patients with angioedema had higher CRP (*p* = 0.038) and UAS7 scores (*p* < 0.001). **Conclusions:** CU exhibits marked immunometabolic heterogeneity. Elevated DAO and lactic acid indicate increased histamine turnover and metabolic activation, whereas altered sCD14-normalized biomarker profiles reveal immune dysregulation in anti-TPO-positive patients. Severe CU with features suggestive of thyroid autoimmunity manifests as a mast cell-dominant, metabolically active phenotype with relative suppression of innate immune modulators, contrasting with alternative pathways in other CU phenotypes. The lactic acid/DAO ratio may serve as a candidate biomarker of disease control. These results underscore the importance of phenotype-tailored therapeutic strategies in CU.

## 1. Introduction

Chronic urticaria (CU) is a common and clinically heterogeneous inflammatory skin disorder that markedly impairs patients’ quality of life [1]. CU comprises multiple endotypes driven by distinct yet overlapping immunopathogenic mechanisms [2,3,4]. Although mast cell activation represents the final common pathway underlying wheal formation, increasing evidence suggests that CU arises from a complex interplay among innate immune activation, autoimmunity, and systemic inflammatory processes. Notably, a substantial proportion of CU cases are considered autoimmune in nature, characterized by the presence of autoreactive antibodies and impaired immune tolerance [4].

The intestinal epithelial barrier plays a pivotal role in regulating host–microbial interactions, maintaining immune homeostasis, and supporting systemic metabolic balance [5]. Disruption of this barrier has been implicated in a broad spectrum of disorders, including autoimmune, metabolic, neuropsychiatric, hepatic and allergic diseases [6,7,8,9,10,11,12,13,14,15]. Alterations in barrier integrity, commonly referred to as “leaky barrier”, may facilitate the translocation of microbial components and metabolites into the systemic circulation, thereby promoting immune activation and metabolic reprogramming. In addition, dietary factors, probiotics, and microbiota-derived metabolites can modulate barrier function, enhancing epithelial resilience and influencing mast cell activity and innate immune responses [7,11,16].

Growing attention has been directed toward the gut–skin axis as a potential contributor to inflammatory skin diseases. Emerging evidence suggests bidirectional communication between the intestinal barrier and the skin, mediated by (i) systemic dissemination of microbial metabolites; (ii) translocation of microbial components, such as endotoxins, into the circulation; and (iii) microbiota-mediated modulation of systemic immune responses. These mechanisms have been implicated in dermatologic conditions including atopic dermatitis, psoriasis, and acne, supporting the concept that intestinal homeostasis may influence cutaneous inflammation [6,11,12].

In CU, however, the role of gastrointestinal (GI) barrier integrity and GI-associated inflammation remains incompletely understood. Although several studies have reported alterations in gut microbiota composition in CU, mechanistic evidence linking intestinal barrier dysfunction to disease activity remains limited. Available data suggest that patients with CU exhibit reduced gut microbial beta diversity, decreased circulating short-chain fatty acids (SCFAs), known inhibitors of mast cell activation, and microbiome alterations associated with inflammatory burden, disease duration, and treatment response. These findings raise the possibility that intestinal dysregulation may contribute to mast cell activation, metabolic reprogramming, and systemic immune imbalance in CU [17,18,19,20,21,22,23,24,25,26].

Importantly, most previous investigations have primarily focused on microbial composition rather than functional markers of epithelial barrier integrity or innate immune activation. Whether GI epithelial permeability, microbial translocation, and systemic innate immune mediators such as soluble CD14 are associated with disease severity, circulating histamine levels, antihistamine resistance, or autoimmune features remains unclear. Given the heterogeneity of CU, we hypothesized that GI barrier dysfunction and low-grade intestinal inflammation may contribute to distinct immunologic phenotypes, particularly in patients with autoimmune features. Accordingly, the primary aim of this study was to evaluate markers of GI epithelial barrier integrity and inflammation in patients with CU and to examine their relationships with systemic inflammatory markers, disease severity, circulating histamine levels, antihistamine resistance, and autoimmune status.

## 2. Materials and Methods

### 2.1. Study Population

Patients who were either newly diagnosed or under follow-up at the Dermatoallergy Unit and fulfilled the diagnostic criteria for CU based on routine clinical and laboratory evaluation were enrolled. The study protocol was approved by the Institutional Research Ethics Committee (Approval No: 24-8T/107). Informed consent was obtained from all participants.

Demographic and clinical characteristics, including age, sex, urticaria subtype (spontaneous, inducible, or combined), disease severity (Urticaria activity score 7 (UAS7) ≥ 28 defined as severe), disease control status (Urticaria Control Test (UCT) < 12 defined as uncontrolled), presence of GI symptoms and angioedema, antihistamine resistance, anti-thyroid peroxidase (anti-TPO) positivity, and laboratory parameters (serum IgE, C-reactive protein [CRP], neutrophil, lymphocyte, eosinophil, and basophil counts, neutrophil-to-lymphocyte ratio [NLR], eosinophil-to-lymphocyte ratio [ELR]) were recorded. Anti-TPO positivity was considered an indicator of underlying thyroid autoimmunity rather than a definitive marker to define autoimmune CU. IgE levels > 100 kU/L were classified as elevated.

A total of 40 patients with CU and 40 healthy controls were included in the study. The control group was matched to the patient group in terms of age and sex and was recruited either from individuals presenting to the dermatology outpatient clinic for localized, non-systemic conditions or from hospital staff who agreed to participate.

Participants were excluded if they had a history of infection within the previous 3 months, mastocytosis or mast cell activation syndrome, malignancy, hematologic, lymphoproliferative, or inflammatory GI diseases, or if they had received antibiotics, systemic corticosteroids, immunosuppressive therapies, or nonsteroidal anti-inflammatory drugs within the previous 3 months, or probiotics within the previous month. Additional exclusion criteria included pregnancy, lactation, age under 18 years, and major surgery within the previous month. Patients who had received omalizumab therapy prior to blood sampling were excluded from the study. In antihistamine-resistant patients, blood samples were obtained before the initiation of omalizumab treatment.

### 2.2. Sample Collection and Processing

Peripheral venous blood samples were collected into serum separator tubes (yellow cap, gel-containing) using Vacutainer^®^ (Disera, İzmir, Türkiye). systems. Samples were stored at 4–8 °C and centrifuged within 6 h at 2000× *g* for 10 min. Serum was aliquoted and stored at −80 °C until analysis. Frozen serum samples were thawed at room temperature under controlled conditions and briefly centrifuged prior to assay. All pre-analytical procedures were standardized to minimize variability.

### 2.3. ELISA Measurements

Serum levels of GI epithelial barrier dysfunction markers, zonulin, intestinal fatty acid-binding protein (IFABP), soluble CD14 (sCD14), diamine oxidase (DAO), D-lactic acid, and endotoxin, were measured. Calprotectin was assessed as a marker of mucosal inflammation, and serum histamine levels were determined.

Sandwich ELISA kits were used to measure IFABP (E-EL-H0159), DAO (E-EL-H1241), calprotectin (E-EL-H2357), zonulin (E-EL-H5560), and sCD14 (E-EL-H6149; Elabscience, Houston, TX, USA), as well as endotoxin (E1801Hu) and D-lactic acid (BT-E4380Hu) (Bioassay Technology Laboratory, Shanghai, China). Histamine levels were determined using a competitive ELISA kit (E-EL-0032; Elabscience, Houston, TX, USA), according to the manufacturers’ instructions. Samples were diluted (1:10, 1:50, or 1:100) according to preliminary optimization experiments. All samples and standards were measured in triplicate. For sandwich ELISA, optical density (OD) values were converted to concentrations using standard curves. For competitive ELISA, OD values were inversely proportional to histamine concentration.

To evaluate interactions between barrier dysfunction, inflammation, and histamine metabolism, the following ratios were calculated: Histamine/DAO, D-lactic acid/DAO, D-lactic acid/Histamine, D-lactic acid/sCD14, D-lactic acid/Endotoxin, D-lactic acid/Calprotectin, IFABP/sCD14, Zonulin/sCD14, Endotoxin/sCD14, and Calprotectin/sCD14.

### 2.4. Additional Analyses

In addition to ELISA parameters, demographic variables, clinical characteristics (disease subtype, severity, control, antihistamine resistance, anti-TPO positivity, presence of angioedema), and laboratory parameters (IgE, CRP, NLR, ELR) were analyzed to explore correlations with epithelial barrier dysfunction, metabolic activity, and mast cell effector mechanisms.

### 2.5. Statistical Analyses

All analyses were performed using IBM SPSS Statistics 25.0. Continuous variables were tested for normality using the Shapiro–Wilk test, with skewness and kurtosis also evaluated. Normally distributed variables were expressed as mean ± standard deviation (SD) and compared with independent samples *t*-tests, while non-normally distributed variables were expressed as median (interquartile range, IQR) and compared with Mann–Whitney U tests. Categorical variables were analyzed using the chi-square (χ^2^) test. Spearman’s rank correlation analysis was used to evaluate associations between parameters. Receiver operating characteristic (ROC) curve analysis was performed to evaluate the discriminatory performance of selected biomarkers and ratios for disease control or severity. Multivariable regression analysis was conducted to identify independent associations between clinical, metabolic, and immunologic variables. A two-tailed *p* value < 0.05 was considered statistically significant.

## 3. Results

### 3.1. Patient Characteristics

A total of 40 patients with CU and 40 healthy controls were included. The mean age was 40.45 ± 15.4 years in the CU group and 37.80 ± 13.57 years in controls (*p* = 0.547), with comparable sex distribution (female/male: 29/11 vs. 27/13, *p* = 0.626).

Within the urticaria cohort, 31 patients (77.5%) had severe disease, and 22 (55%) exhibited antihistamine resistance. Spontaneous urticaria was observed in 32 patients (80%), inducible urticaria in 5 patients (12.5%), and both forms in 3 patients (7.5%). Angioedema was present in 15 patients (37.5%). Elevated IgE levels were observed in 23 patients (59%). Disease control was achieved in 5 patients (12.5%), while 8 patients (20%) had both severe and anti-TPO-positive urticaria (Table 1).

### 3.2. Biomarker Profiles

**Figure 1:**
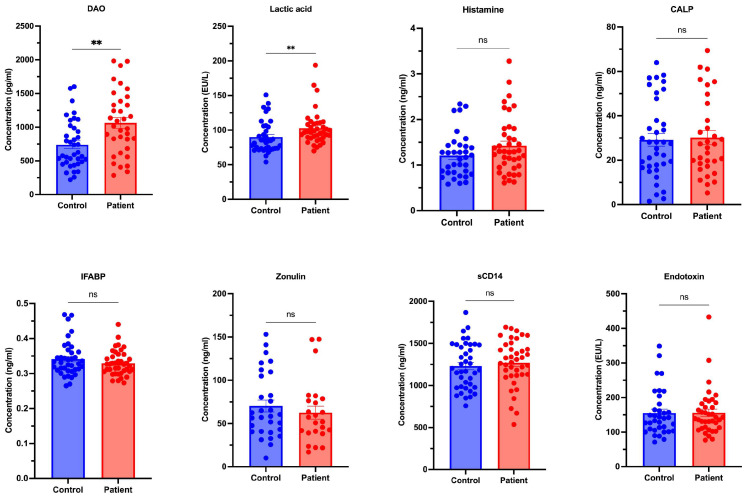
Comparison of ELISA Parameters Between Chronic Urticaria and Control Groups. ** indicates statistical significance (*p* < 0.05).

Biomarker analysis revealed significantly higher DAO (*p* = 0.003) and lactic acid (*p* = 0.004) in CU patients compared with controls, whereas zonulin, IFABP, sCD14, endotoxin, and calprotectin showed no significant differences (Figure 1, Table 2).

In anti-TPO-positive patients, sCD14 levels were significantly lower (*p* = 0.024), while the ratios of histamine/sCD14 (*p* = 0.005), lactic acid/sCD14 (*p* = 0.014), IFABP/sCD14 (*p* = 0.008), and zonulin/sCD14 (*p* = 0.027) were significantly higher, suggesting relative amplification of metabolic and barrier-associated pathways under CD14-mediated regulatory deficiency.

Comparing severe anti-TPO-positive with severe anti-TPO-negative cases, sCD14 (*p* = 0.022) and NLR (*p* = 0.013) were lower, whereas UAS7 (*p* = 0.032), histamine (*p* = 0.011), calprotectin (*p* = 0.041), and CD14-normalized ratios—including histamine, IFABP, lactic acid, zonulin, and calprotectin—were significantly higher, indicating a mast cell-dominant, metabolically active inflammatory phenotype in autoimmune CU.

According to the UCT, the lactic acid/DAO ratio was significantly lower in controlled versus uncontrolled patients (*p* = 0.013), while other parameters did not differ. This ratio was associated with uncontrolled disease in univariate analysis and showed good discriminatory performance in ROC analysis, though it lost significance in multivariable regression, suggesting it reflects overall disease activity rather than an independent pathogenic pathway.

No significant differences in biomarkers were observed between antihistamine-resistant and antihistamine-responsive patients. In addition, no differences were observed between the spontaneous and inducible forms in any parameter. Patients with angioedema exhibited higher CRP (*p* = 0.038) and UAS7 (*p* < 0.001) compared with those without angioedema, while other biomarkers were comparable. The presence of GI symptoms was not associated with any ELISA parameters (all *p* > 0.05).

When patients with severe urticaria (UAS7 ≥ 28) were compared with those with mild-to-moderate urticaria, serum sCD14 (*p* = 0.021), lactic acid/DAO (*p* = 0.042), lactic acid/histamine (*p* = 0.012), and histamine/DAO (*p* = 0.006) were significantly higher in the severe urticaria group. In contrast, IFABP/CD14 values were significantly lower (*p* = 0.034). No significant differences were observed in the other parameters or their ratios (Table 3).

## 4. Discussion

**Figure 2:**
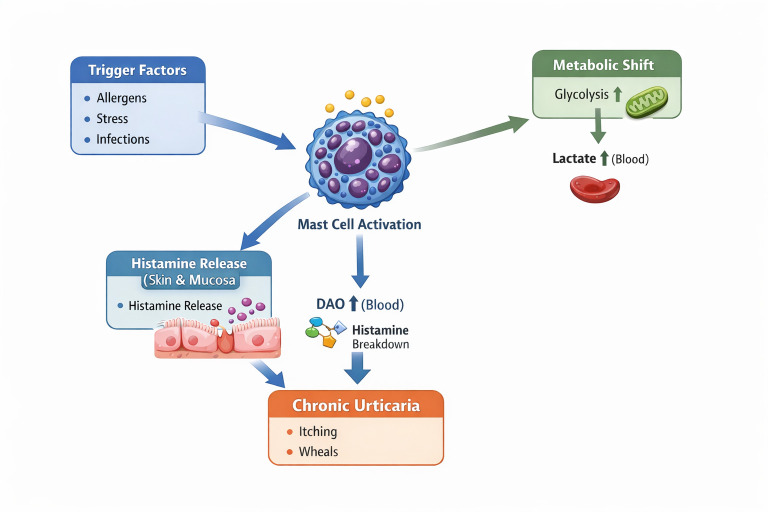
Proposed mechanistic model of mast cell activation and metabolic shift in chronic urticaria.

In the present study, patients with CU exhibited significantly elevated serum lactic acid and DAO levels compared with healthy controls, while markers of epithelial damage (IFABP), barrier dysfunction (zonulin), microbial translocation (endotoxin), and inflammation (calprotectin) were not significantly different. These findings suggest that CU represents a metabolically active yet partially regulated inflammatory state, in which DAO upregulation may function as a compensatory mechanism to buffer increased histamine burden generated from chronic mast cell activation [27,28,29] (Figure 2). Although histamine levels were elevated, the absence of statistically significant differences indicates that histamine alone cannot fully explain the pathophysiology, emphasizing the contribution of metabolic reprogramming and innate immune regulation. Future studies with larger, well-characterized cohorts and standardized measurement approaches are warranted to clarify the clinical relevance of these biomarkers and to better define their potential roles in disease mechanisms in CU.

Analysis of disease control revealed that the lactic acid/DAO ratio was significantly lower in controlled patients compared with uncontrolled cases, suggesting that the balance between metabolic activity and histamine degradation may reflect the biological state of disease control. Although elevated lactic acid levels may partially reflect a general inflammatory state, the lactic acid/DAO ratio may provide additional insight into disease control beyond classical inflammatory markers. Therefore, while the lactic acid/DAO ratio may serve as a useful indicator of disease control, these findings should be interpreted within the context of overall systemic immune and metabolic activity, rather than as a disease-specific mechanism [27,28]. Furthermore, the lack of linear correlation between lactic acid and UAS7 in severe cases suggests a threshold-dependent model of inflammatory activation, in which metabolic and immune responses may be triggered only after a critical level of inflammatory burden is reached. The lower IFABP/sCD14 ratio in severe CU further indicates that systemic immune activation, rather than overt intestinal epithelial damage, predominates in disease progression.

Soluble CD14 (sCD14) represents a key regulator at the interface between innate immune sensing and inflammatory signaling and has been associated with autoimmune and inflammatory conditions, including rheumatoid arthritis [30]. In our study, severe urticaria was associated with elevated sCD14, lactic acid/DAO, lactic acid/histamine, and histamine/DAO ratios, suggesting that both innate immune activation and metabolic reprogramming become more pronounced with disease severity. Interestingly, in the anti-TPO-positive subgroup, sCD14 levels were significantly lower, whereas histamine/CD14, lactic acid/CD14, IFABP/CD14, and zonulin/CD14 ratios were elevated. This pattern suggests a relative amplification of metabolic and barrier-associated signals in the context of deficient innate immune regulation, indicating that CSU with underlying thyroid autoimmunity (as reflected by anti-TPO positivity) is characterized more by dysregulated immunomodulation than by absolute inflammation.

**Figure 3:**
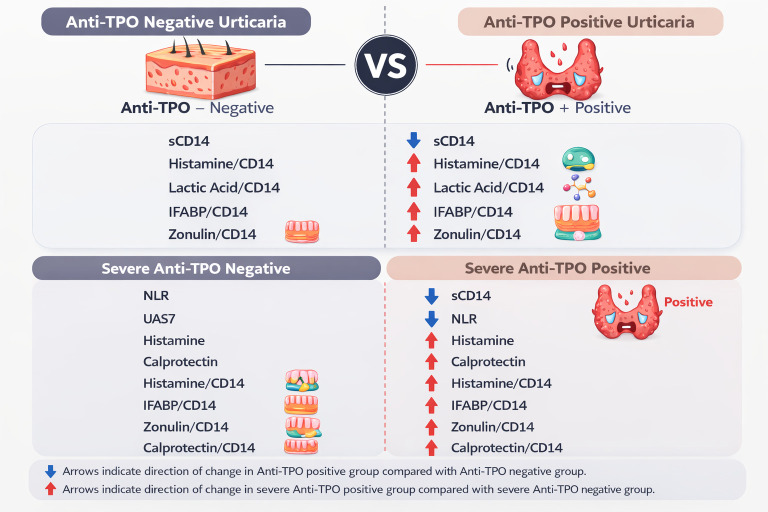
Comparison of biomarker profiles between anti-TPO-positive and anti-TPO-negative patients with chronic urticaria.

Comparison of severe anti-TPO-positive versus anti-TPO-negative patients revealed that CD14 and NLR were significantly lower in the autoimmune group, while UAS7, histamine, calprotectin, and CD14-normalized ratios were higher (Figure 3). This pattern is consistent with a mast cell-dominant, metabolically active, lymphocyte/adaptive immune-predominant inflammatory phenotype, contrasting with anti-TPO-negative patients in whom classical neutrophilic or IgE-mediated pathways may predominate. The observation of elevated calprotectin despite low NLR suggests that tissue-level neutrophil activation or secondary inflammatory amplification mechanisms may operate independently of peripheral neutrophil counts. Collectively, these findings emphasize the immunological heterogeneity of CU, with innate and adaptive immune mechanisms contributing to disease pathophysiology in varying proportions depending on the phenotype.

Of note, the presence of GI symptoms was not correlated with biomarker levels, suggesting that subclinical changes in gut permeability and barrier dysfunction may occur without overt GI manifestations. This highlights the possibility that gut-derived immune activation may contribute to systemic inflammation even in the absence of clinical enteric symptoms, and that GI symptom assessment alone may be insufficient for predicting immune-metabolic disturbances.

This study has some limitations. The relatively small sample size and the inclusion of a small subset of patients with inducible and mixed urticaria may have introduced clinical heterogeneity. In addition, the cross-sectional design and the limited specificity of the evaluated biomarkers may restrict their clinical applicability. Therefore, larger, well-designed studies are needed to confirm these findings.

## 5. Conclusions

This study provides evidence that CU is immunologically and metabolically heterogeneous, with distinct phenotypic patterns shaped by mast cell activity, metabolic alterations, innate immune modulation, and barrier-associated mechanisms. Patients with features suggestive of thyroid autoimmunity, partly reflected by anti-TPO positivity, showed enhanced metabolic and mast cell-related responses, along with evidence of innate immune dysregulation. However, anti-TPO positivity alone may not fully capture the complexity of autoimmune CU. In contrast, other phenotypic subsets may involve alternative inflammatory pathways.

The results underscore the potential of lactic acid/DAO and sCD14-normalized ratios as biomarkers for disease control and immune-metabolic balance, although their clinical utility requires further validation. These observations may have implications for personalized therapeutic strategies; however, phenotype-driven treatment approaches should be interpreted with caution. Future prospective studies are warranted to validate these phenotypes and to investigate their relationship with treatment response, ultimately facilitating more precise management of CU. 

## Figures and Tables

**Table 1:** Demographic and clinical characteristics of patients with chronic urticaria.

Parameter	Values
Age (mean ± SD)	40.45 ± 15.4
Sex (F/M)	29/11
Chronic urticaria subtype, *n* (%)	
Spontaneous	32 (80)
Inducible	5 (12.5)
Spontaneous + inducible	3 (7.5)
Presence of angioedema, *n* (%)	15 (35.7)
Antihistamine resistance, *n* (%)	22 (55)
Anti-TPO positivity, *n* (%)	12 (30.8)
Elevated IgE, *n* (%)	23 (59)
Elevated CRP, *n* (%)	11 (27.5)
UAS7 (mean ± SD)	31.25 ± 12.25
UCT score (mean ± SD)	6.3 ± 4.24

**Table 2:** Comparison of ELISA parameters between chronic urticaria and control groups.

Parameter	Control	Chronic Urticaria	*p*
Histamine (ng/mL), median (IQR)	1.17 (0.6)	1.30 (0.8)	0.122
DAO (pg/mL), median (IQR)	609.4 (521.6)	1014.8 (702.9)	**0** **.003**
sCD14 (ng/mL) *, mean ± SD	1230.74 ± 271.74	1269.97 ± 277.43	0.525
Lactic acid (EU/L), median (IQR)	85.5 (31.2)	96.7 (14.6)	**0** **.004**
Endotoxin (EU/L), median (IQR)	143.9 (52.5)	147.5 (53.6)	0.711
IFABP (ng/mL), median (IQR)	0.34 (0.06)	0.32 (0.05)	0.346
Zonulin (ng/mL), median (IQR)	56.5 (30.6)	51.4 (38.9)	0.497
Calprotectin (ng/mL), median (IQR)	21.2 (19.4)	25.3 (40.5)	0.861
Histamine/DAO, median (IQR)	0.0015 (0.0014)	0.0011 (0.0011)	0.3
Lactic acid/DAO, median (IQR)	0.1 (0.08)	0.10 (0.08)	0.052

Data are presented as mean ± SD for normally distributed variables * and median (interquartile range, IQR) for non-normally distributed variables. *p* values were calculated using independent samples *t*-test * or Mann–Whitney U test as appropriate. DAO, diamine oxidase; SD, standard deviation; IQR, interquartile range. Statistically significant *p*-values are presented in bold font.

**Table 3:** Comparison of parameters between patients with severe urticaria and those with mild-to-moderate urticaria.

Parameter	Mild-Moderate CU	Severe CU	*p* Value
Histamine (ng/mL), mean ± SD	1.16 ± 0.69	1.50 ± 0.60	0.156
DAO (pg/mL), mean ± SD	1314.03 ± 513.74	976.66 ± 434.39	0.064
sCD14 (ng/mL), mean ± SD	1065.73 ± 319.67	1329.26 ± 238.03	**0.021**
Lactic acid (EU/L), mean ± SD	101.35± 7.64	103.16 ± 27.35	0.856
Endotoxin (EU/L), mean ± SD	146.28 ± 47.52	158.50 ± 70.58	0.648
IFABP (ng/mL), mean ± SD	0.33 ± 0.023	0.33± 0.04	0.828
Zonulin (ng/mL), mmean ± SD	69.28 ± 55.21	59.85 ± 27.29	0.679
Calprotectin (ng/mL), mean ± SD	28.40 ± 19.86	30.85± 17.05	0.30
Histamine/DAO *, median (IQR)	0.0008 (0.0005)	0.0016 (0.0021)	**0** **.006**
Lactic acid/DAO *, median (IQR)	0.08 (0.05)	0.11 (0.14)	**0** **.042**
Lactic acid/Histamine *, median (IQR)	118.14 (67.7)	72.97 (46.95)	**0.012**
IFABP/sCD14 *, median (IQR)	0.00029 (0.00024)	0.00024 (0.00008)	**0.034**

Data are presented as mean ± SD for normally distributed variables and median (interquartile range, IQR) * for non-normally distributed variables. *p* values were calculated using independent samples *t*-test or Mann–Whitney U test * as appropriate. CU, chronic urticaria; DAO, diamine oxidase; SD, standard deviation; IQR, interquartile range; IFABP, Intestinal Fatty Acid-Binding Protein. Statistically significant *p*-values are presented in bold font.

## Data Availability

The data presented in this study are available from the corresponding author upon reasonable request. The data are not publicly available due to privacy and ethical restrictions.

## Abbreviations

The following abbreviations are used in this manuscript:

DAO	Diamine oxidase
IFABP	Intestinal fatty acid-binding protein
sCD14	Soluble CD14
UAS7	Urticaria Activity Score 7
UCT	Urticaria Control Test
CRP	C-reactive protein
NLR	Neutrophil-to-lymphocyte ratio
ELR	Eosinophil-to-lymphocyte ratio
GI	Gastrointestinal
ELISA	Enzyme-linked immunosorbent assay
IQR	Interquartile range
SD	Standard deviation
